# Editorial on the Special Issue “Novel Methods of Diagnostics of Thyroid and Parathyroid Lesions”

**DOI:** 10.3390/jcm11040932

**Published:** 2022-02-11

**Authors:** Ewelina Szczepanek-Parulska, Marek Ruchala

**Affiliations:** Department of Endocrinology, Metabolism and Internal Medicine, Poznan University of Medical Sciences, 61-701 Poznan, Poland; mruchala@ump.edu.pl

Thyroid nodular disease is one of the most frequent endocrine diseases. The prevalence of thyroid focal lesions detected by imaging techniques according to studies on different populations ranges from 10 to 70% [1,2]. In a population of women over 50 years of age, approximately half of them would have a thyroid focal lesion. This, in turn, poses an important diagnostic dilemma, as only the minority (3–15%) of such lesions exhibit malignancy [3]. The standard investigations of the thyroid nodules consist of ultrasonography coupled with qualification of the lesions for cytological examination. The pivotal role of ultrasound examination is to estimate the risk of malignancy and to select lesions for a fine-needle aspiration biopsy (FNAB). The results of cytological assessment directly determine the decision regarding the treatment. Unfortunately, both conventional ultrasonography and biopsy have certain limitations. Moreover, the evaluation of an increasing number of thyroid incidentalomas constitutes a huge burden on the healthcare system. The performance of conventional ultrasound in the differentiation of thyroid nodules is moderate, with a sensitivity equal to 68–100% and specificity ranging within 67–94%. It must be followed by a FNAB—an invasive procedure yielding inconclusive results in approximately 15–30% (3/4 of them will eventually prove to be benign on histopathology). In 2015 >600,000 of FNABs were performed in the USA [4]. Hence, there is a need for a non-invasive technique which would help to reliably assess the thyroid nodules malignancy risk. High hopes have been pinned on sonoelastography and the application of artificial intelligence [5]. In addition, molecular studies have become an increasingly useful and available tool in thyroid cancer risk assessment [6,7]. In particular, we hope that the introduction of these methods will allow us to reduce the amount of invasive practices performed unnecessarily in patients with benign nodules. It is also very important to evaluate whether the actual clinical practice guidelines are followed and how this influences the proper qualification of patients for thyroidectomy [8]. On the other hand, the techniques of surgical management are being constantly improved (mini-invasive procedures, monitoring of laryngeal nerve), which contributes to better outcomes, reduces the number of complications, and results in better post-surgery quality of life for the patients [9].

Another frequent endocrine disease that poses both a diagnostic and therapeutic challenge is primary hyperparathyroidism (PHP) [10,11,12]. It is diagnosed most frequently in women during the perimenopausal age, while male patients are affected four times less frequently [13]. However, when the underlying genetic cause such as MEN syndrome is present, PHP might also be diagnosed much earlier in life [14]. The incidence in the USA is approximated at 0.86% of the population [15], while the estimations for normocalcemic hyperparathyroidism vary widely according to different studies, and ranges from 0.4 to 11%; however, some of those cases are supposed to be related to vitamin D deficiency as opposed to classical PHP. Nevertheless, it is obvious that in developed countries, the diagnostic rate of PHP has increased over the past several decades. Better access to both biochemical evaluation and imaging diagnostics might contribute to that fact [13]. Better recognition of PHP is related to better diagnostics of localisation of the primary lesion, which improves the outcomes and enables a mini-invasive approach during parathyroidectomy.

The aim of this review is to present the current challenges and novel methods of diagnostics and management of thyroid nodular disease and PHP, with a particular emphasis on the issues described in the papers from the Special Issue entitled “Novel Methods of Diagnostics of Thyroid and Parathyroid Lesions”, published by the *Journal of Clinical Medicine*.

In the study by Adamczewski et al., a possible impact of the probe plane (longitudinal vs. transverse) on the elasticity values measured by ShearWave Elastography (SWE) method was evaluated [16]. In the study, the SWE technique was shown to present high intra- and interobserver agreement for the measurement of elastic properties of ectopic thymus (ET) and surrounding thyroid parenchyma. Important statistical differences were noted between obtained elasticity values, but not for relative elasticity scores. The authors explained this phenomenon by possible anisotropy-related artifacts; however, they also stated that the observed differences do not significantly affect the reliability of the method. The authors concluded that awareness of the existence of plane-dependent differences is crucial for proper interpretation of the measured values.

In another paper, the same group demonstrates the applicability of the SWE technique for differentiation between papillary thyroid cancer (PTC) and ET in children [17]. The group studied consisted of 31 subjects, in whom 53 foci of ETs were depicted. The authors expressed the elasticity with a quantitative method. The results demonstrated that the elasticity of ETs is similar or even higher compared to that of the thyroid gland, while SWE proved to be a valuable technique for diagnostics of ET cases. The authors concluded that SWE might allow us to avoid the biopsy at least in some cases, which at the same time should undergo a careful follow-up involving conventional ultrasound examination coupled with SWE assessment.

In the prospective study by Szczepanek-Parulska et al., the potential to differentiate the character of thyroid lesions of new proposed ultrasound-based methods is validated—EU-TIRADS classification and commercially available software S-Detect, and a combinations of both [1]. In the study, 88 patients were included who had been diagnosed with 133 focal lesions, and who were later on subjected to thyroidectomy and/or FNAB. Only those patients in whom unambiguous results of histopathological or cytological examination were obtained were qualified for analysis. S-detect software turned out to be a highly sensitive method, presenting good specificity. The classification of nodules with the use of EU-TIRADS scale turned out to be highly sensitive, though not very specific. The best results in terms of diagnostic performance of malignant lesions were obtained for the S-Detect (when it provided estimation of the “possibly malignant” nodule), while, simultaneously, the lesion gained four or five points or exactly five points in EU-TIRADS scale.

The study by Wieczorek-Szukala et al. encompassed 61 patients diagnosed with PTC, who also underwent BRAF^V600E^ mutation status evaluation [18]. Their results demonstrated that the Snail-1expression correlated with the metastatic potential of PTC. Such an observation was not confirmed with regard to TGFβ1. It was evidenced for the first time that upregulation of Snail-1 corresponded to the presence of BRAF^V600E^ mutation. In addition, authors provided evidence that overexpression of Snail-1 is dependent on BRAF^V600E^ mutation status. In another study, Jensen et al., with the use of microfluidic digital PCR and co-amplification at lower denaturation temperature (COLD) PCR, evaluated the cell-free DNA with *BRAFV600E* mutations (cf*BRAFV600E*) obtained from peripheral blood. The 57 samples from patients diagnosed with PTC harboring somatic *BRAFV600E* mutation were studied [19]. The cf DNA with *BRAFV600E* mutation was found in 42.1% of the studied subjects, while its detection has been demonstrated to correlate with the size of the primary tumor, multifocal form, extrathyroidal spread of the disease and lack of excellent response to treatment if compared to patients with undetectable mutated cf DNA. The studied method has been recently successfully introduced for the evaluation of patients with neoplasms. The authors conclude that the tested method is useful to identify patients presenting higher probability of non-excellet response to therapy, either biochemical or structural, might not be excellent.

The study by Castellnou et al. analyzed the influence of the presurgical management on appropriate evaluation of indications for surgery [20]. The source of data was a nationwide cohort study on patients operated on between 2012 and 2015. Three pathways were distinguished: (1) “FNAB—containing only FNAB, (2) “FNAB+ENDO”—including a consultation by an endocrinologist, (3) “NO FNAB”—FNAB was not performed. Among the 1080 patients, 18.2% underwent “FNAB+ENDO”, 19.2%—“FNAB”, while 62.6% “NO FNAB”. Comparing to “NO FNAB”, “FNAB+ENDO” care pathway more frequently was associated with a diagnosis of thyroid cancer (OR 2.67, 1.88–3.81), which was similar to “FNAB” path (OR 2.09, 1.46–2.98). Following thyroidectomies, which were performed at university hospitals, a thyroid cancer diagnosis was established more often (OR 1.61, 1.19–2.17). The recommended care pathway was found to provide more relevant estimation of indications for surgery. The authors noted that although guidelines regarding pre-surgical procedures had not been satisfactorily put into practice, the adherence to recommendations had improved throughout the years.

In another paper, the advantages of the application of intraoperative neuromonitoring of the recurrent laryngeal nerve (IONM) were shown, which, according to authors’ findings, reduces the rate of one of the most important complications of thyroid surgeries in bilateral planned thyroidectomies —bilateral vocal cord dysfunction (bVCD) [21]. This retrospective analysis involved prospectively documented data from 20-year experience of a tertiary referral center. Out of 22,573 patients involved in the analysis, only 65 developed bVCD (0.288%). The rate of bVCD was 0.44 prior to the introduction of the routine use of the procedure, and dropped to 0.09% after routine implementation of IONM (*p* < 0.001). In conclusion, taking into consideration the presented results, the authors state that IONM should be recommended for planned and bilateral thyroidectomies. Non-complete bilateral surgery rate after intraoperative non-transient loss of signal in the studied group reached 2%.

In the paper by Smaxwill et al., the potential to identify parathyroid adenomas (PAs) of [^18^F]fluoro-ethylcholine-PET-CT&4D-CT (FEC-PET&4D-CT) was retrospectively analysed in subjects, in whom ultrasound or MIBI-scintiscan failed to provide direct localisation. In 171 FEC-PET&4D-CTs, the presence of PAs was suggested in 159 of examinations (92.9%). Among 147 patients who already had parathyroidectomy, FEC-PET&4D-CT precisely identified PA in 141 (false negative results in four patients, false positive in two patients). The sensitivity reached 97% and the accuracy reached 96%, while the positive predictive value (PPV) was equal to 99%. Thus, authors conclude that FEC-PET&4D-CT shows exceptional correctness in localization of PAs, where traditional methods are not able to provide accurate localisation [22].

The study by Hung et al. evaluated the role of ultrasound-guided parathormone (PTH) concentration assessment in neck lesions suspected of being PAs in a group of subjects with complicated recurrent or persistent secondary hyperparathyroidism [23]. The authors proposed a new definition for a positive result—PTH washout concentration higher than one-thirtieth of the serum PTH. In the study, 32 patients were included, in whom 50 PTH aspirate concentrations were measured. One-third of 39 washout-positive lesions were not visible on scintigraphic examination. A total of 28 lesions (71.8%) had equivocal ultrasonography results. When the results of histopathological examination following surgical treatment were treated as reference, the PTH aspirate concentration measurement characterized with a 100% PPV. Therefore, the authors suggested that PTH concentration tissue aspirate assessment with the proposed new definition of a positive assay could be very useful to localize PAs in subjects suffering from complicated recurrent or persistent hyperparathyroidism of renal origin.

Interesting findings were also presented by Gawrychowski et al. in a study concerning surgical management of PHP [24]. This was a retrospective clinicopathologic analysis on 1019 patients from a single institution treated throughout a period of 35 years. Treatment failure was observed in 19 cases (1.9%); however, the repeated surgical intervention allowed for achieving remission of the disease in 16 cases. The authors conclude that the ectopic mediastinal location of the PA increases the risk of surgical treatment failure. However, the authors also underline that a majority of PAs of mediastinal localization can still be managed from cervical access.

In conclusion, sonoelastography proves to be a useful method in non-invasive differentiation of thyroid focal lesions in children and adult populations. Simultaneous application of both evaluation of thyroid lesions using EU-TIRADS scale and software based on artificial intelligence yielded the best diagnostic performance in malignancy risk prediction. In terms of progress in evaluation of molecular markers of thyroid cancer, the expression of Snail-1 correlates with the metastatic potential of PTC. Determination of the presence of cell-free DNA harboring *BRAFV600E* mutation in samples obtained from the plasma of patients with PTC using microfluidic digital PCR and COLD PCR provides important information for the prediction of response to therapy and metastatic potential of PTC. If surgical therapy is concerned, an appropriate pathway of selection of patients including FNAB and endocrinologist consultation is necessary to adequately qualify eligible subjects for thyroidectomy. Application of IONM of the recurrent laryngeal nerve may help to decrease the incidence of bilateral vocal cord dysfunction in subjects undergoing planned bilateral thyroidectomies. The progress of localization diagnostics of PAs is achieved by application of [^18^F]fluoro-ethylcholine-PET-CT&4D-CT and PTH concentration assessment in washout from ultrasound-guided biopsy, which allows for precise localisation of PAs in patients, in whom conventional methods (ultrasonography, MIBI-scintiscan) failed to provide unequivocal results. Surgical experience shows that the parathyroidectomy is successful method of therapy in the vast majority of patients. Although ectopic mediastinal location of PAs may increase the risk of treatment failure, the majority of PAs of mediastinal localization still can be successfully managed from the cervical access.
